# Increased Thyroid DPP4 Expression Is Associated With Inflammatory Process in Patients With Hashimoto Thyroiditis

**DOI:** 10.1210/clinem/dgad723

**Published:** 2023-12-21

**Authors:** Xiaohui Wen, Xiaona Chang, Xueqing He, Qingyun Cai, Guang Wang, Jia Liu

**Affiliations:** Department of Otolaryngology, Beijing Chao-Yang Hospital, Capital Medical University, Beijing 100020, China; Department of Endocrinology, Beijing Chao-Yang Hospital, Capital Medical University, Beijing 100020, China; Department of Endocrinology, Beijing Chao-Yang Hospital, Capital Medical University, Beijing 100020, China; Department of Endocrinology, Beijing Chao-Yang Hospital, Capital Medical University, Beijing 100020, China; Department of Endocrinology, Beijing Chao-Yang Hospital, Capital Medical University, Beijing 100020, China; Department of Endocrinology, Beijing Chao-Yang Hospital, Capital Medical University, Beijing 100020, China

**Keywords:** dipeptidyl peptidase-4, Hashimoto thyroiditis, lymphocytes, thyroid destruction

## Abstract

**Context:**

Dipeptidyl peptidase-4 (DPP4) is originally described as a surface protein in lymphocytes. Lymphocyte infiltration and subsequent destruction of thyroid tissue have been considered as the central pathological mechanism in Hashimoto thyroiditis (HT).

**Objective:**

The present study aimed to investigate DPP4 expression in peripheral blood and thyroid tissue in HT patients, and explore the role of DPP4 in the pathophysiological process of HT.

**Methods:**

This case-control study recruited 40 drug-naive HT patients and 81 control individuals. Peripheral blood and thyroid specimens were collected for assessing the expression and activity of DPP4. Moreover, single-cell RNA sequencing (scRNA-seq) analysis of 6 “para-tumor tissues” samples from scRNA-seq data set GSE184362 and in vitro cell experiments were also conducted.

**Results:**

The HT patients had similar DPP4 serum concentration and activity as the controls. However, the expression and activity of DPP4 was significantly increased in the thyroid of the HT group than in the control group. The scRNA-seq analysis showed that DPP4 expression was significantly increased in the HT group, and mainly expressed in T cells. Further in vitro studies showed that inhibition of lymphocyte DPP4 activity with sitagliptin downregulated the production of inflammatory factors in co-cultured thyroid cells.

**Conclusion:**

DPP4 expression was significantly increased in the thyroid of the HT group compared with the control group, and was mainly localized in the lymphocytes. Inhibition of lymphocyte DPP4 activity reduced the production of inflammatory factors in co-cultured thyroid cells. Therefore, inhibition of DPP4 may have a beneficial effect by alleviating inflammatory reactions in HT patients.

Dipeptidyl peptidase-4 (DPP4), also known as CD26, is a 110-kDa cell surface glycoprotein and is widely expressed in multiple tissues and organs within the human body (1). Several studies have demonstrated that the expression and activity of DPP4 varies in patients with multiple autoimmune diseases (2-5). Patients with type 1 diabetes have elevated serum DPP4 activity and reduced DPP4 expression in the lymphocyte membrane (2). And decreased serum concentration and activity of DPP4 have been observed in patients with multiple sclerosis (3). Autoimmune thyroid disease, including Graves disease (GD) and Hashimoto thyroiditis (HT), is one of the most common endocrine diseases (6, 7). Our previous study showed that GD patients had significantly increased serum concentration and activity of DPP4 (4). And the increased serum concentration and activity of DPP4 were positively associated with the severity of hyperthyroidism in GD patients (4). However, the status of DPP4 in HT patients remained unclear.

To date, the majority of existing studies have focused on the expression and activity of DPP4 in serum and peripheral blood lymphocytes in HT patients (8-10). The study by Wang et al showed that serum DPP4 concentration was reduced in HT patients, while a similar concentration and activity of DPP4 were observed between the HT and control groups in the study by Liu et al (8, 9). As an organ-specific autoimmune disease, very little research has addressed DPP4 expression in the thyroid tissue of HT patients. The present study aimed to investigate the DPP4 expression in peripheral blood and thyroid tissue in HT patients, and explore the role of DPP4 in the pathophysiological process of HT.

## Materials and Methods

### Study Design and Participants

**Figure 1.**
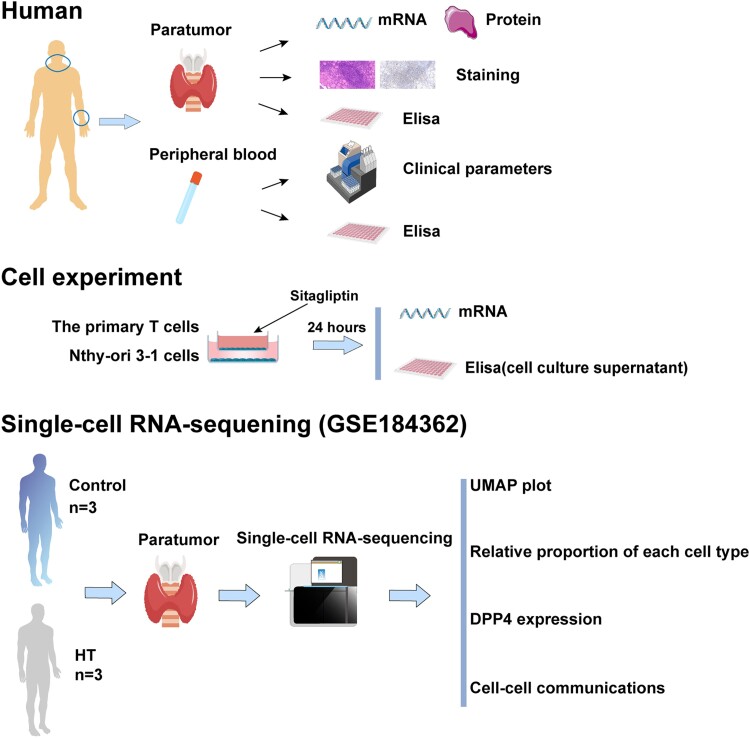
Graphical view of the study road map.

The present study recruited 121 patients who were scheduled for thyroidectomy because of suspicious papillary thyroid carcinoma (<1 cm in diameter) at the Department of Otolaryngology, Beijing Chao-Yang Hospital, Capital Medical University, from June 2021 to December 2022 (Fig. 1). The inclusion criteria for this study were as follows: (1) meeting the surgical indications (2); normal thyroid function (3); receiving neither radiotherapy nor chemotherapy before surgery. No participants had a history of GD, subacute thyroiditis, thyroid dysfunction, diabetes, hypertension, systemic inflammatory disease, infectious diseases, coronary disease, heart failure, liver and renal function impairment, or malignant disease. Participants who ingested agents known to influence thyroid function, glucose, and lipid metabolism were also excluded. All participants were given the following examinations including free triiodothyronine (FT3), free thyroxine (FT4), thyrotropin (TSH), antithyroid peroxidase antibody (TPOAb), antithyroglobulin antibody (TgAb), and thyroid ultrasound. Normal thyroid function was defined when FT3, FT4, and TSH were in the normal ranges (normal range, FT3: 2.63-5.71 pmol/L; FT4: 9.10-19.2 pmol/L; TSH: 0.35-4.94 mIU/L). Positivity for TPOAb or TgAb was diagnosed as a value greater than 60 IU/mL (normal range for TPOAb or TgAb, 0.00∼60.0 IU/mL). The criteria of the control group were as follows: both TPOAb and TgAb were negative; no typical HT pathological characteristics based on thyroid specimens. HT was diagnosed by positive for TPOAb and/or TgAb and typical pathological characteristics with massive lymphocytes infiltration with germinal center formation, and destruction of the follicular structure (11).

The present study complied with the Helsinki Declaration, and the protocol was approved by the ethics committee of the Beijing Chao-Yang Hospital, Capital Medical University. All enrolled participants provided written informed consent.

### Clinical and Biochemical Measurements

Height and weight were measured to the nearest 0.1 cm and 0.1 kg by the same trained group, respectively. Body mass index (BMI) was calculated as weight in kilograms divided by height in meters squared. Meanwhile, information about health status and medications of all participants was collected. Venous blood samples were obtained from all the participants after overnight fasting and before surgery. FT3, FT4, and TSH were measured by electrochemiluminescence immunoassay using an Abbott Architect i2000 (Dimension Vista, Siemens Healthcare Diagnostics). The serum concentrations of TPOAb and TgAb were detected by chemiluminescent immunoassay (Dimension Vista, Siemens Healthcare Diagnostics). White blood cell (WBC), neutrophil, and lymphocyte counts were measured by a Beckman-Coulter Ac.T5Diff hematology analyzer. Alanine transaminase (ALT) and aspartate transaminase (AST) were measured using the velocity method (Hitachi 747, Roche Diagnostics). Glycated hemoglobin A_1c_ (HbA_1c_) was detected by high-performance liquid chromatography using the HLC-723G7 analyzer (Tosoh Corporation). Thyroid ultrasound examination and evaluation were conducted by professional sonographers before operation.

### Measurements of Dipeptidyl Peptidase-4 Concentration and Activity

Serum DPP4 concentration was measured by enzyme-linked immunosorbent assay (ELISA; Nos. DC260B, R&D, RRID: AB_3073870) in accordance with the manufacturer's instructions. DPP4 activity in serum and thyroid tissue was detected using a commercial DPP4 activity assay kit (No. MAK088, Sigma Systems). Thyroid tissue was diluted 50-fold with DPP4 assay buffer, homogenized, and the supernatant was used to detect DPP4 activity. DPP4 cleaves a nonfluorescent substrate, H-Gly-Pro-AMC, to release a fluorescent product, 7-amino-4-methyl coumarin (AMC) (λex = 360/λem = 460 nm). One unit of DPP4 is the amount of enzyme that will hydrolyze the DPP4 substrate to yield 1.0 μmol of AMC per minute at 37 °C.

### Thyroid Specimens and Staining

Thyroid specimens were collected from at least 2 cm away from the nodule. Part of thyroid tissue specimen was stored at −80 °C within 30 minutes for total RNA extraction. Part of thyroid tissue specimen was fixed in 10% formalin to make paraffin-embedded blocks further and finally subjected to hematoxylin and eosin staining and immunohistochemistry (IHC) staining according to a standard protocol. Thyroid sections were incubated with primary antibody against DPP4 (ab215711, Abcam, RRID: AB_2734752) and IHC Detection Reagent (PV-8000, ZSGB-BIO). Histological images of tissue sections were taken with a light microscope (Nikon Eclipse Ni).

### Culture of Cells

The human thyroid follicular epithelial (Nthy-ori-3-1) cell line was obtained from the European Collection of Animal Cell Cultures (ECACC). The primary T cells obtained by EasySep Human T Cell Isolation Kit (catalog No. 17951, STEMCELL) from human peripheral blood mononuclear cell were used as co-cultured T lymphocytes. The primary T cells and Nthy-ori-3-1 cells were all cultured in RPMI-1640 (Gibco) containing 10% (v/v) fetal bovine serum (Gibco), 1% (v/v) penicillin streptomycin solution (Invitrogen), incubated at 37 °C in a humidified atmosphere of 5% CO_2_. To activate T cells, 1 μg/mL of Human CD3/CD28 T Cell Activators (catalog Nos. 317325 and 302933, BioLegend) were added to cell culture medium as well as to expend T cells, 0.1 mg/mL Human Recombinant IL-2 (catalog No. 78036, STEMCELL) was added.

Co-culture of Nthy-ori 3-1 cells and primary T cells mimicked the pathological environment of HT. The co-culture environment was established by adding primary T cells to the upper chamber of a Transwell 6-well plate and Nthy-ori 3-1 cells to the lower chamber of a Transwell 6-well plate. The primary T cells were treated with or without sitagliptin (300 μM) for 24 hours. Then Nthy-ori 3-1 cells were collected to detect the messenger RNA (mRNA) expression levels of interleukin 18 (IL-18), interleukin 1β (IL-1β), and interleukin 6 (IL-6). Additionally, the concentration of interferon γ (IFN-γ) and IL-6 in the cell culture supernatant from the lower chamber of a Transwell 6-well plate was detected.

### Total RNA Extraction and Real-time Polymerase Chain Reaction

Total RNA was extracted from thyroid tissue or Nthy-ori-3-1 cells using the TRIzol (T9424, Sigma) method. The RNA concentration was measured using a Nano Drop spectrophotometer (Thermo Fisher Scientific) to ensure that the optical density of 260/280 was between 1.8 and 2.0. Complementary DNA was obtained by HiScript III All-in-One RT SuperMix Kit (R333, Vazyme) according to the manufacturer's instructions. Quantitative real-time polymerase chain reaction (qRT-PCR) was performed using ChamQ Universal SYBR qPCR Master Mix (Q331, Vazyme) in a 7500-detection system (Applied Biosystems). Relative gene expression (normalized to the endogenous control gene β-actin) was calculated using the ^ΔΔ^Ct method. RT-PCR primer sequences for human β-actin, IFN-γ, tumor necrosis factor α (TNF-α), IL-1β, IL-6, IL-18, and DPP4 are listed in Supplementary Table 1 (12).

### Total Protein Extraction and Western Blot Analysis

Thyroid tissue was homogenized in lysate supplemented with protease inhibitors and phosphatase inhibitors, and total protein was collected after centrifugation. Protein concentration was measured with the BCA protein assay kit (Thermo Scientific). Tissue lysates were mixed with sodium dodecyl sulfate sample buffer, boiled, and separated by 8% sodium dodecyl sulfate–polyacrylamide gel electrophoresis. The proteins were then electrotransferred to polyvinylidene difluoride (PVDF) membranes, which were incubated overnight at 4 °C with anti-DPP4 antibody (ab215711, Abcam, RRID: AB_2734752). Glyceraldehyde-3-phosphate dehydrogenase (GAPDH) antibody (10494-1-AP, Proteintech, RRID: AB_2263076) was used as control. PVDF membranes were then incubated with peroxidase-conjugated secondary antibodies, and specific bands were detected with a Bio-Rad imaging system. Band intensity was measured using ImageJ software.

### Enzyme-linked Immunosorbent Assay

The concentrations of IFN-γ and IL-6 in the culture supernatant were detected by ELISA using commercial kits (IL-6: D6050, R&D, RRID: AB_2928038; IFN-γ: ab46025, Abcam, RRID: AB_3073869). The absorbance of all samples was measured at 450 nm, 540 nm, or 620 nm using a microplate reader (Thermo Fisher Scientific). According to the absorbance values and the gradient concentration of the standard product provided by the kit, the standard curve of 4 parameter logistic (4-PL) curve fitting was generated.

### Single-Cell RNA Sequencing Data Collection

The single-cell RNA sequencing (scRNA-seq) data set GSE184362 of papillary thyroid carcinoma was obtained from the Gene Expression Omnibus (GEO, https://www.ncbi.nlm.nih.gov/geo/), of which 6 “para-tumor tissues” samples were selected for subsequent analysis (Fig. 1) (13). According to whether complicated by HT, the 6 samples were split into 2 groups: a control group (CT group: n = 3), and an HT group (n = 3). The clinical characteristics of these participants, including thyroid function, neoplasm staging, and pathological features, are shown in Supplementary Table 2 (12).

### Statistical Analysis

Differences of clinical parameters and cell experiments were analyzed using SPSS 26.0 (SPSS). The differences between the 2 groups were analyzed using an independent-sample *t* test or a Mann-Whitney *U* test. The differences among multiple groups were analyzed by analysis of variance test or Kruskal-Wallis *H* test followed by Bonferroni post hoc tests. The proportions were analyzed using chi-square tests. Correlation analyses were performed using Pearson and Spearman correlations. Statistical significance was considered with 2-tailed analyses as *P* less than .05. scRNA-seq data analysis was conducted using R software (v.4.2.3). Further details about the procedure, including cell filtering, quality control, cell type clustering, annotation, and cell-cell communication, are provided in the supplementary materials (12).

## Results

### Baseline Characteristics of the Control and Hashimoto Thyroiditis Groups

**Figure 2.**
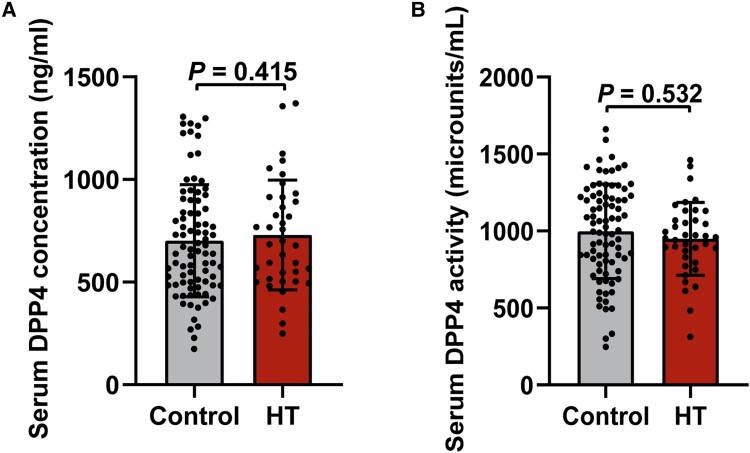
The serum concentration and activity of DPP4 in the control and HT groups. A, Serum DPP4 concentration. B, Serum DPP4 activity. Data are shown as mean ± SD. DPP4, dipeptidyl peptidase-4; HT, Hashimoto thyroiditis.

The baseline characteristics of the CT and HT groups are shown in Table 1. No statistically significant differences in age, sex, BMI, FT3, FT4, or TSH levels were observed between the two groups. The levels of WBCs, neutrophil, and lymphocyte were also statistically the same between the control and HT groups. The HT patients had similar ALT, AST, and HbA_1c_ levels when compared with the CTs. The levels of TPOAb and TgAb were significantly increased in the HT group compared with the CT group (*P* < .001; see Table 1). However, there was no significant difference in the serum concentration and activity of DPP4 between the HT and CT groups (see Table 1 and Fig. 2). Although we collected thyroid tissue from at least 2 cm away from the papillary thyroid microcarcinoma, it may not totally eliminate the influence of the tumor. Further subgroup analyses were performed to compare the DPP4 expression between patients with and without papillary thyroid microcarcinoma within the groups. The results demonstrated that there was no significant difference in the serum DPP4 concentration/activity and the DPP4 mRNA expression/activity of thyroid between benign thyroid nodules and papillary thyroid carcinoma arms in the two groups (Supplementary Tables 3 and 4) (12).

**Table 1.** Baseline characteristics of the control and Hashimoto thyroiditis groups

Parameters	Control	HT	*P*
	(n = 78)	(n = 43)	
Age, y	45 ± 11	44 ± 12	.854
Sex, male/female, n	20/58	6/37	.134
BMI	25.00 ± 4.23	24.76 ± 3.87	.759
FT3, pmol/L	5.05 ± 0.49	4.87 ± 0.50	.068
FT4, pmol/L	15.51 ± 1.94	15.04 ± 1.95	.203
TSH, mIU/mL	1.75 (1.31-2.47)	1.86 (1.28-2.91)	.254
TPOAb, IU/mL	28 (28-32.9)	141.2 (28-1850.6)	<.001
TgAb, IU/mL	15 (15-18.1)	141.9 (40.2-262.2)	<.001
WBC, 10^9^/L	6.70 ± 1.66	6.85 ± 1.49	.627
Neutrophil, 10^9^/L	4.06 ± 1.36	4.24 ± 1.27	.496
Lymphocyte, 10^9^/L	2.10 ± 0.52	2.09 ± 0.72	.942
ALT, U/L	16 (12-23)	16 (13-22)	.770
AST, U/L	18 (16-22)	19 (17-23)	.426
HbA_1c_, %	5.5 ± 0.3	5.4 ± 0.4	.322
Serum DPP4 concentration, ng/mL	699.20 ± 276.28	738.63 ± 268.46	.454
Serum DPP4 activity, microunits/mL	997.69 ± 302.99	959.12 ± 245.53	.481

Normally distributed variables were expressed as mean ± SD, while variables with a skewed distribution were expressed as the median and upper and lower quartiles. Abbreviations: ALT, alanine transaminase; AST, aspartate transaminase; BMI, body mass index; DPP4, dipeptidyl peptidase-4; FT3, free triiodothyronine; FT4, free thyroxine; HbA_1c_, glycated hemoglobin A_1c_; HT, Hashimoto thyroiditis; TgAb, antithyroglobulin antibodies; TPOAb, antithyroid peroxidase antibodies; TSH, thyrotropin; WBC, white blood cell.

### Expression of Dipeptidyl Peptidase-4 in Thyroid Tissue of Control and Hashimoto Thyroid Groups

**Figure 3.**
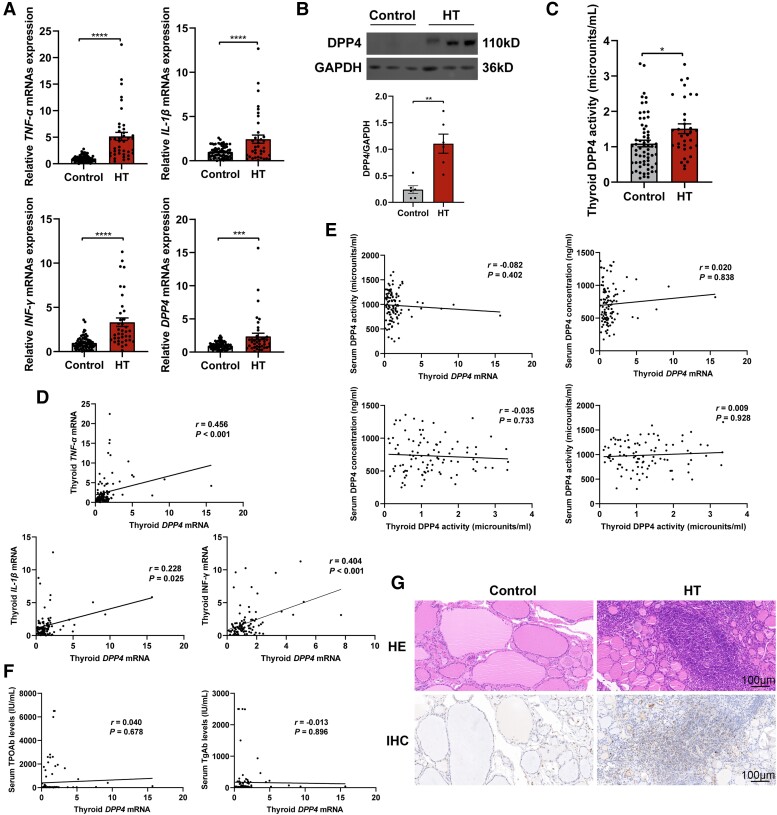
Thyroid DPP4 expression and its association with inflammatory factors. A, mRNA expression of DPP4 and inflammatory factors in the thyroid of the control and HT groups. B, The protein expression of thyroid DPP4 in the thyroid of the control and HT groups. GAPDH was used as a loading control. C, The DPP4 activity in the thyroid of the control and HT groups. D, Correlation analysis of the DPP4 mRNA expression and the mRNA expression of inflammatory factors in thyroid tissue. E, Correlation analysis between the mRNA expression and activity of DPP4 in thyroid tissue and the serum concentration and activity of DPP4. F, Correlation analysis between the mRNA expression of DPP4 and serum TPOAb/TgAb levels. G, Enhanced DPP4 expression on lymphocytes in thyroid tissue of HT patients. Representative images of HE and IHC staining of DPP4 in the thyroid sections from control and HT patients (200× magnification, light microscope). Scale bar = 100μm. Data are shown as mean ± SEM. *P less than .05, **P less than .01, ***P less than .001. DPP4, dipeptidyl peptidase-4; HE, hematoxylin and eosin staining; HT, Hashimoto thyroiditis; GAPDH, glyceraldehyde-3-phosphate dehydrogenase; IHC, immunohistochemistry; IL-1β, interleukin 1β; INF-γ, interferon γ; mRNA, messenger RNA; TNF-α, tumor necrosis factor α.

As shown in Fig. 3A, the mRNA expression of IL-1β, TNF-α, IFN-γ, and DPP4 was significantly increased in the thyroid of HT patients compared with CTs (all *P* < .001). Compared with the CT group, the thyroid DPP4 protein levels of the HT patients were also markedly elevated (*P* < .01; Fig. 3B). Consistently, enhanced DPP4 activity was observed in the thyroid tissues of HT patients when compared with the CTs (Fig. 3C). Subsequent correlation analysis showed that the thyroid DPP4 mRNA expression was positively associated with the inflammatory factors IL-1β, TNF-α, and IFN-γ (Fig. 3D). However, there was no significant correlation between the thyroid DPP4 expression/activity and the serum DPP4 concentration/activity (Fig. 3E). No significant association between DPP4 expression and TPOAb/TgAb titers was observed (Fig. 3F). IHC staining showed that expression of DPP4 was notably increased in the HT patients, and mainly localized in the lymphocytes (Fig. 3G). In contrast, DPP4 expression was only rarely observed in the thyroid tissue of the CT participants (Fig. 3G).

### Expression Level of Dipeptidyl Peptidase-4 in Single-Cell RNA Sequencing Data

**Figure 4.**
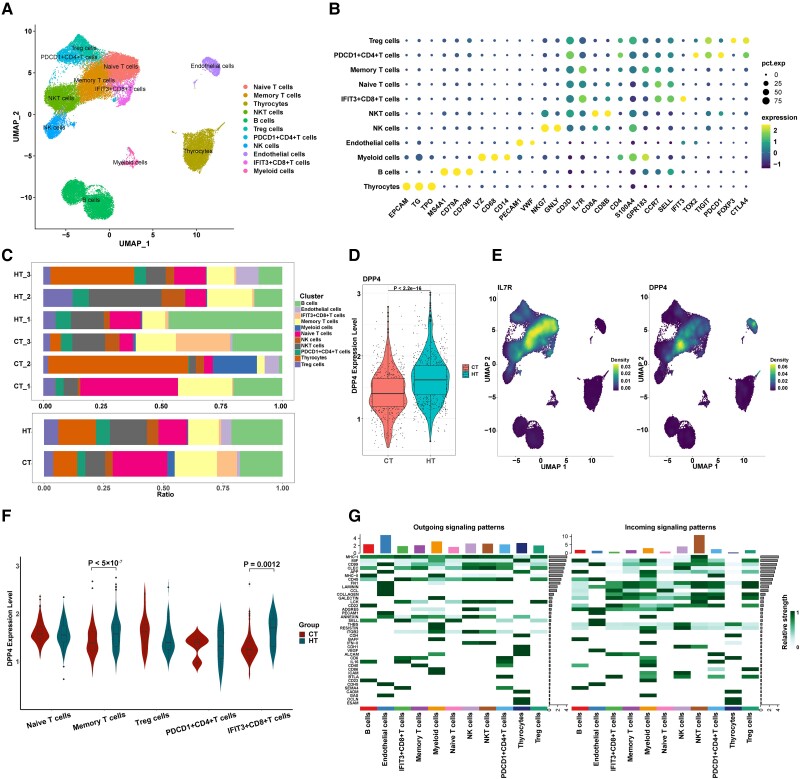
Overview of single-cell transcriptomic analysis. A, UMAP plot of the 11 identified cell types. B, Dot plots showing the 28 marker gene expressions among the 11 cell types. The size of dots represents the proportion of cells expressing the particular marker gene, and the color spectrum indicates the mean expression levels of the marker gene (log transformed). C, Relative proportion of each cell type. D, The DPP4 expression in the CT and HT group. E, UMAP plot highlighting the gene expression of IL7R (T cell) and DPP4. F, DPP4 expression in different T-cell cluster between HT and control groups. G, The cell-cell communications heat map based on the relative expression of ligand-receptor pairs. CT, control group; DPP4, dipeptidyl peptidase-4; HT, Hashimoto thyroiditis; UMAP, uniform maximal approximation projection.

After strict quality control filtration and batch correction, a total of 45 802 cells were included in the subsequent analysis (Supplementary Table 5).(12) Unsupervised clustering analysis identified 19 clusters, and 11 cell categories were annotated, including thyrocytes, B cells, myeloid cells, endothelial cells, natural killer cells, natural killer T cells, IFIT3 + CD8+ T cells, naive T cells, memory T cells, PDCD1 + CD4+ T cells, and regulatory T cells, according to the expression of canonical markers and the most variable genes (Fig. 4A and Supplementary Table 5) (12-17). Fig. 4B shows the expression dot plot of marker genes. The cell proportions of each sample and cell cluster are presented in Fig. 4C. As expected, the DPP4 expression level of the HT group was significantly increased when compared with the CT group (*P* < .0001; Fig. 4D). DPP4 is displayed visually in the cell cluster expression diagram (Fig. 4E). Interestingly, T cells, especially memory T cells, had significantly higher DPP4 expression among the 11 cell types (Fig. 4E). The expression levels of DPP4 in memory T cells and IFIT3 + CD8+ T cells in HT patients was significantly higher than that in CTs (Fig. 4F). We further analyzed the outgoing and incoming signal pathways based on the relative expression of ligand-receptor pairs and found that memory T cells engaged in a range of functional interactions mediated by CD40, CD45, L-selectin (SELL), and major histocompatibility complex class I molecules (MHC-I), signaling that is mainly involved in the inflammatory response (Fig. 4G).

### Inhibition of Dipeptidyl Peptidase-4 Exerted Anti-inflammatory Properties

**Figure 5.**
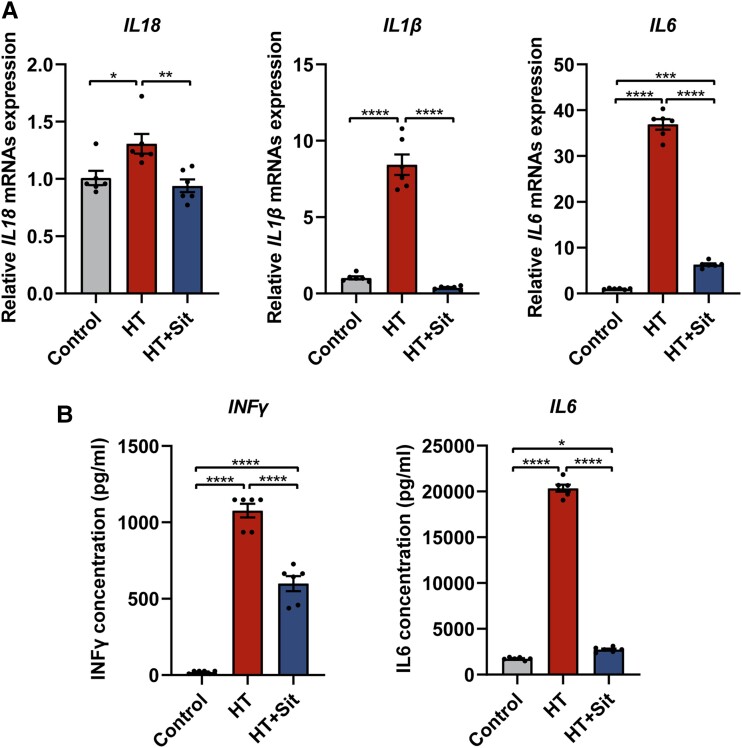
Inflammatory factor expression of Nthy-ori 3-1 cells and cell culture supernatant at co-culture environment. The co-culture environment was established by adding primary T cells to the upper chamber of a Transwell 6-well plate and Nthy-ori 3-1 cells to the lower chamber of a Transwell 6-well plate in the HT group and HT + Sit group. Cell culture medium was placed to the upper chamber of a Transwell 6-well plate as the vehicle control. A, The mRNA expression of IL-18, IL-1β, and IL-6 in Nthy-ori 3-1 cells. B, The concentration of INF-γ and IL-6 of the cell culture supernatant in the lower chamber of a Transwell 6-well plate. Data are shown as mean ± SEM. *P less than .05, **P less than .01, ***P less than .001. IL-1β, interleukin 1β; IL-6, interleukin 6; IL-18, interleukin 18; INF-γ, interferon γ; mRNA, messenger RNA; HT, Hashimoto thyroiditis; Sit: sitagliptin.

As DPP4 was mainly expressed in lymphocytes, further studies were conducted to investigate the effect of sitagliptin, a kind of DPP4 inhibitor, in a co-culture environment of primary T cells and Nthy-ori 3-1 cells. The primary T cells elevated the mRNA expression of IL-18, IL-1β, and IL-6 in the co-culture Nthy-ori 3-1 cells (Fig. 5A). Interestingly, inhibition of DPP4 activity in T cells with sitagliptin downregulated the mRNA expression of the aforementioned inflammatory factors in co-cultured Nthy-ori 3-1 cells (Fig. 5A). Additionally, the changes of cytokines in cell culture supernatants were also consistent (Fig. 5B).

## Discussion

Previous studies have indicated that the expression and activity of DPP4 changes in patients with several autoimmune diseases (2-5). The present study showed that the mRNA and protein expression of DPP4 was significantly increased in the thyroid of the HT group compared with that of the CT group. Enhanced DPP4 activity was also observed in the thyroid of the HT patients. IHC staining showed that expression of DPP4 was notably increased in HT patients. Consistently, scRNA-seq analysis showed that DPP4 expression was significantly increased in the HT group compared with the CT group. The correlation analysis showed that in thyroid tissue, DPP4 mRNA was positively related to the mRNA expression of many inflammatory factors, including IL-1β, TNF-α, and IFN-γ. Therefore, the results suggested that the increased DPP4 expression in thyroid tissue might be associated with the pathogenesis of HT.

Consistent with many previous studies, IHC staining in the present study found that DPP4 expression was mainly localized in the lymphocytes (18, 19). Meanwhile, the results mentioned earlier were confirmed by the scRNA-seq analysis, which indicated that DPP4 was mainly expressed in T cells, especially memory T cells. The outgoing and incoming signal pathway analysis indicated that memory T cells engaged in a range of functional interactions mediated by CD40, CD45, SELL, and MHC-I signaling that were mainly involved in the inflammatory response. As we know, DPP4 was originally described as a surface protein in lymphocytes and plays an important role in the maturation, differentiation, and activation of lymphocytes, and participates in immune regulation (20, 21). As an organ-specific autoimmune disease, lymphocyte infiltration and subsequent destruction of thyroid tissue had been considered as the central pathological mechanism in HT (22, 23). Infiltrated T lymphocytes promote the expression of inflammatory factors, including IL-1β, TNF-α, and IFN-γ, and further cause destruction of follicular structure in HT patients (23, 24). Therefore, a further in vitro experiment was conducted to investigate whether DPP4 inhibitors could alleviate the inflammatory state of HT. Interestingly, inhibition of lymphocyte DPP4 activity with sitagliptin downregulated the production of inflammatory factors in co-cultured thyroid cells. In agreement with the present study, some previous studies have shown that inhibition of DPP4 reduced IL-2 and IFN-γ production and suppressed T-cell proliferation in antigen-stimulated T cells (25, 26). Moreover, DPP4 inhibitor reduced T-cell infiltration in islets, alleviated insulitis, and decreased the development of type 1 diabetes in nonobese diabetic mice (27). The DPP4 inhibiter sitagliptin decreased the expression of the proinflammatory cytokine in patients with type 2 diabetes (28). Furthermore, a recent meta-analysis found that DPP4 inhibitor administration was associated with a decreased risk of rheumatoid arthritis (29). Based on this evidence, the increased DPP4 expression of T cells might be linked with inflammation and the immune process in thyroid tissue, and DPP4 inhibition may have a beneficial effect by alleviating inflammatory reactions in HT patients.

Moreover, as an organ-specific autoimmune disease, the immunoinflammatory responses were mainly concentrated in the thyroid tissue of HT patients (7, 11). However, the decreased serum DPP4 concentration was observed in HT patients in a small-scale study with 20 healthy controls and 31 HT patients by Wang et al (8). Additionally, research has shown that no significant differences in serum DPP4 levels were identified between an HT group and a CT group (30). So, we further compared the serum concentration and activity of DPP4 between HT and CT groups. The present study was consistent with the study by Liu et al (9) and found that there was no significant difference in serum concentration and activity of DPP4 between the HT and CT groups. Further correlation analysis indicated that the expression and activity of DPP4 in thyroid tissue did not relate to the serum concentration and activity of DPP4, which might be explained by the organ-specific properties of HT. Several studies have demonstrated that circulating soluble DPP4 is generated by many cell types, and is influenced by body weight, glucose metabolism, WBC, and hepatic function (18, 31, 32, 33). In the present study, there was no significant difference in age, sex, BMI, HbA_1c_, WBC, and hepatic function between the HT and CT groups. Unfortunately, the study by Wang et al (8) did not display the levels of BMI, HbA_1c_, WBC, or hepatic function of the HT and CT groups, so the inconsistency might be associated with the aforementioned confounding factors.

The present study has several limitations. First, this study was a single-center study with a relatively small sample size, thus, it might introduce some confounders that influenced the results. Further studies with a large sample size are required to confirm our results. Second, previous studies have found that DPP4 expression is decreased in certain periphery T-cell subsets in HT patients compared with healthy CTs, while Wang et al thought that the percentages of CD26/DPP4 expression on Tc1, Tc2, Th1, and Th2 cells were not different between healthy CTs and HT patients (8, 9). An explanation for this difference could be that thyroid function affects metabolism, which further alters DPP4 expression (34-36). Since HT is an organ-specific autoimmune disease, we mainly focused on the DPP4 expression of thyroid tissue in HT patients with normal thyroid function; the DPP4 expression of peripheral blood lymphocytes was not addressed. Finally, we did not investigate the effects of DPP4 inhibitors in the experimental autoimmune thyroiditis (EAT) mice model. Further research is needed to better understand the role of DPP4 in the pathogenesis of HT and to elucidate the precise mechanisms for the upregulation of DPP4 expression.

In conclusion, the DPP4 expression was significantly increased in the thyroid of the HT group compared with the CT group, and was mainly localized in the lymphocytes. Inhibition of lymphocyte DPP4 activity reduced the production of inflammatory factors in co-cultured thyroid cells. Therefore, DPP4 inhibition may have a beneficial effect by alleviating inflammatory reactions in HT patients.

## Data Availability

All data for this study are available from the corresponding authors on reasonable request.

## Abbreviations

ALT: alanine transaminase
AMC: 7-amino-4-methyl coumarin
AST: aspartate transaminase
BMI: body mass index
CT: control
DPP4: dipeptidyl peptidase-4
ELISA: enzyme-linked immunosorbent assay
FT3: free triiodothyronine
FT4: free thyroxine
GD: Graves disease
HbA_1c_: glycated hemoglobin A_1c_
HT: Hashimoto thyroiditis
IL-1β: interleukin 1β
IL-6: interleukin 6
IL-18: interleukin 18
INF-γ: interferon γ
qRT-PCR: quantitative reverse-transcription polymerase chain reaction
scRNA-seq: single-cell RNA sequencing
TgAb: antithyroglobulin antibodies
TNF-α: tumor necrosis factor α
TPOAb: antithyroid peroxidase antibodies
TSH: thyrotropin
WBC: white blood cell

## References

[1] Ansorge S, Nordhoff K, Bank U, et al Novel aspects of cellular action of dipeptidyl peptidase IV/CD26. Biol Chem. 2011;392(3):153‐168. 21194362 10.1515/BC.2011.008

[2] Varga T, Somogyi A, Barna G, et al Higher serum DPP-4 enzyme activity and decreased lymphocyte CD26 expression in type 1 diabetes. Pathol Oncol Res: POR. 2011;17(4):925‐930. 21785903 10.1007/s12253-011-9404-9

[3] Tejera-Alhambra M, Casrouge A, de Andrés C, et al Low DPP4 expression and activity in multiple sclerosis. Clin Immunol (Orlando, Fla). 2014;150(2):170‐183. 10.1016/j.clim.2013.11.011 24412911

[4] Chang X, Ding X, Wang J, Cai Q, Wang G, Liu J. The serum concentration and activity of DPP4 is positively related with the severity of hyperthyroidism in patients with Graves’ disease. Ann Med. 2023;55(1):2226910. 37350750 10.1080/07853890.2023.2226910 PMC10291917

[5] Watanabe T, Temma Y, Okada J, et al In patients with type 2 diabetes the presence of Hashimoto's thyroiditis reduces the beneficial effect of dipeptidyl peptidase-4 inhibitor on plasma glucose control. Endocr J. 2021;68(5):599‐603. 33408313 10.1507/endocrj.EJ20-0620

[6] Smith TJ, Hegedüs L. Graves’ disease. N Engl J Med. 2016;375(16):1552‐1565. 27797318 10.1056/NEJMra1510030

[7] Weetman AP . An update on the pathogenesis of Hashimoto's thyroiditis. J Endocrinol Invest. 2021;44(5):883‐890. 33332019 10.1007/s40618-020-01477-1 PMC8049926

[8] Wang Z, Yang Y, Liu S, et al CD26/DPP4 levels in peripheral blood and T cells in Hashimoto's thyroiditis with normal thyroid function. Int Immunopharmacol. 2019;77:105941. 31670093 10.1016/j.intimp.2019.105941

[9] Liu Y, Li Y, Gong Y, et al CD26 expression is down-regulated on CD8+ T cells in patients with Hashimoto's thyroiditis. Int Immunopharmacol. 2018;54:280‐285. 29175506 10.1016/j.intimp.2017.11.024

[10] Xu W, Liu Y, Cheng X, et al Decreased shedding dipeptidyl peptidase 4 from membrane in Hashimoto's thyroiditis. Int Immunopharmacol. 2020;81:106315. 32086131 10.1016/j.intimp.2020.106315

[11] Ragusa F, Fallahi P, Elia G, et al Hashimotos’ thyroiditis: epidemiology, pathogenesis, clinic and therapy. Best Pract & Res Clin Endocrinol & Metabol. 2019;33(6):101367. 10.1016/j.beem.2019.101367 31812326

[12] Wen X, Chang X, He X, et al Supplementary data for: “Increased thyroid DPP4 expression is associated with inflammatory process in patients with Hashimoto’s thyroiditis”. J Clin Endocrinol Metab. Deposited November 10, 2023. 10.1210/clinem/dgad723 PMC11099486 38127960

[13] Pu W, Shi X, Yu P, et al Single-cell transcriptomic analysis of the tumor ecosystems underlying initiation and progression of papillary thyroid carcinoma. Nat Commun. 2021;12(1):6058. 34663816 10.1038/s41467-021-26343-3 PMC8523550

[14] Wang T, Shi J, Li L, et al Single-Cell transcriptome analysis reveals inter-tumor heterogeneity in bilateral papillary thyroid carcinoma. Front Immunol. 2022;13:840811. 35515000 10.3389/fimmu.2022.840811 PMC9065345

[15] Zhou Y, Yang D, Yang Q, et al Single-cell RNA landscape of intratumoral heterogeneity and immunosuppressive microenvironment in advanced osteosarcoma. Nat Commun. 2020;11(1):6322. 33303760 10.1038/s41467-020-20059-6 PMC7730477

[16] Perez RK, Gordon MG, Subramaniam M, et al Single-cell RNA-seq reveals cell type-specific molecular and genetic associations to lupus. Science (New York, NY). 2022;376(6589):eabf1970. 10.1126/science.abf1970 PMC9297655 35389781

[17] Zhang J-Y, Wang X-M, Xing X, et al Single-cell landscape of immunological responses in patients with COVID-19. Nat Immunol. 2020;21(9):1107‐1118. 32788748 10.1038/s41590-020-0762-x

[18] Casrouge A, Sauer AV, Barreira da Silva R, et al Lymphocytes are a major source of circulating soluble dipeptidyl peptidase 4. Clin Exp Immunol. 2018;194(2):166‐179. 30251416 10.1111/cei.13163 PMC6194339

[19] Kholová I, Ryska A, Ludvíková M, Cáp J, Pecen L. Dipeptidyl peptidase IV expression in thyroid cytology: retrospective histologically confirmed study. Cytopathology. 2003;14(1):27‐31. 12588307 10.1046/j.1365-2303.2003.01138.x

[20] Hosono O, Ohnuma K, Dang NH, Morimoto C. CD26: a key molecule in immune regulation and autoimmune diseases. Modern Rheumatol. 2003;13(3):199‐204. 10.3109/s10165-003-0224-y 24387205

[21] Zhao Y . CD26 in autoimmune diseases: the other side of “moonlight protein”. Int Immunopharmacol. 2019;75:105757. 31357088 10.1016/j.intimp.2019.105757

[22] Antonelli A, Ferrari SM, Corrado A, Di Domenicantonio A, Fallahi P. Autoimmune thyroid disorders. Autoimmun Rev. 2015;14(2):174‐180. 25461470 10.1016/j.autrev.2014.10.016

[23] Pyzik A, Grywalska E, Matyjaszek-Matuszek B, Roliński J. Immune disorders in Hashimoto's thyroiditis: what do we know so far? J Immunol Res. 2015;2015:979167. 26000316 10.1155/2015/979167 PMC4426893

[24] Guo Q, Wu Y, Hou Y, et al Cytokine secretion and pyroptosis of thyroid follicular cells mediated by enhanced NLRP3, NLRP1, NLRC4, and AIM2 inflammasomes are associated with autoimmune thyroiditis. Front Immunol. 2018;9:1197. 29915579 10.3389/fimmu.2018.01197 PMC5994487

[25] Flentke GR, Munoz E, Huber BT, Plaut AG, Kettner CA, Bachovchin WW. Inhibition of dipeptidyl aminopeptidase IV (DP-IV) by Xaa-boroPro dipeptides and use of these inhibitors to examine the role of DP-IV in T-cell function. Proc Natl Acad Sci USA. 1991;88(4):1556‐1559. 1671716 10.1073/pnas.88.4.1556 PMC51058

[26] Schön E, Demuth HU, Eichmann E, et al Dipeptidyl peptidase IV in human T lymphocytes. Impaired induction of interleukin 2 and gamma interferon due to specific inhibition of dipeptidyl peptidase IV. Scand J Immunol. 1989;29(2):127‐132. 2564215 10.1111/j.1365-3083.1989.tb01108.x

[27] He X, Li W, Xie Y, Zhao Y. Long-term inhibition of dipeptidyl-peptidase 4 reduces islet infiltration and downregulates IL-1β and IL-12 in NOD mice. Int Immunopharmacol. 2020;88:106945. 33182020 10.1016/j.intimp.2020.106945 PMC7510641

[28] Makdissi A, Ghanim H, Vora M, et al Sitagliptin exerts an antinflammatory action. J Clin Endocrinol Metab. 2012;97(9):3333‐3341. 22745245 10.1210/jc.2012-1544 PMC3431580

[29] Charoenngam N, Rittiphairoj T, Ponvilawan B, Ungprasert P. Use of dipeptidyl peptidase-4 inhibitors is associated with a lower risk of rheumatoid arthritis in patients with type 2 diabetes mellitus: a systematic review and meta-analysis of cohort studies. Diabetes Metab Syndr. 2021;15(1):249‐255. 33465685 10.1016/j.dsx.2020.12.042

[30] Zhang Y, Fu Y, Yang Y, Ke J, Zhao D. Assessment of serum dipeptidyl peptidase-IV levels in autoimmune thyroid disease. J Int Med Res. 2022;50(7):3000605221112031. 10.1177/03000605221112031 PMC9340981 35903860

[31] Lamers D, Famulla S, Wronkowitz N, et al Dipeptidyl peptidase 4 is a novel adipokine potentially linking obesity to the metabolic syndrome. Diabetes. 2011;60(7):1917‐1925. 21593202 10.2337/db10-1707 PMC3121429

[32] Balaban YH, Korkusuz P, Simsek H, et al Dipeptidyl peptidase IV (DDP IV) in NASH patients. Ann Hepatol. 2007;6(4):242‐250. 18007554

[33] Gorrell MD . Dipeptidyl peptidase IV and related enzymes in cell biology and liver disorders. Clin Sci (London, England: 1979). 2005;108(4):277‐292. 10.1042/CS20040302 15584901

[34] Rai C, Priyadarshini P. Whey protein hydrolysates improve high-fat-diet-induced obesity by modulating the brain-peripheral axis of GLP-1 through inhibition of DPP-4 function in mice. Eur J Nutr. 2023;62(6):2489‐2507. 37154934 10.1007/s00394-023-03162-4

[35] Barchetta I, Cimini FA, Dule S, Cavallo MG. Dipeptidyl peptidase 4 (DPP4) as A novel adipokine: role in metabolism and fat homeostasis. Biomedicines. 2022;10(9):2306. 36140405 10.3390/biomedicines10092306 PMC9496088

[36] Park H-K, Ahima RS. Endocrine disorders associated with obesity. Best Pract Res Clin Obstet Gynaecol. 2023;90:102394. 37523934 10.1016/j.bpobgyn.2023.102394

